# The Best Form of Medical Service

**Published:** 1921-01-29

**Authors:** H. L. Croxley


### THE EDITOR'S LETTER-BOX.

**Correspondence on all subjects is invited, but we cannot in any way be responsible for the opinions expressed by our correspondents, who must give their name and address as a guarantee of good faith, but not necessarily for publication. Correspondents are reminded that brevity of style and conciseness of statement greatly facilitate early insertion.**

### THE BEST FORM OF MEDICAL SERVICE.

### Some Views from the General Public.

*To the Editor of* THE HOSPITAL.

SIR,—Your extremely well-reasoned leading article in the issue of THE HOSPITAL for January 22 moves me to write that the lay support accorded any particular social reform is to a large extent guided by the knowledge gained from similar measures of uplift already in vogue, and it is also substantially moulded by the breathings in the Press. If the thought of his previous experience of Government health control is sufficient to make a person feel ill, then, under the guidance of his daily newspaper, he may easily be put to bed.

Anyway, the trenchant recommendation that first ushered in the National Insurance Act—*i.e.* it would assist a class of the community which generally is forgetful to make sufficient provision for itself—will not cover the enlarged scope suggested by the Minister of Health; and it is from the proposed new recruits to National Insurance that some of the severest criticism may be expected.

When the average person feels indisposed, he prefers to attend the doctor whom he imagines can treat him with the greatest proficiency. If his medical adviser wishes to supply a certain kind of medicine, he hates to know that any power on earth can prevent his having that particular tonic, because it is not as prescribed "according to the Act." In a small way his mind is developed by the personality of the medicine man, and it is controlled, in a larger way, by hearsay. Thus, in our villages, if Mrs. B. has been cured by Dr. Q. of severe indigestion, the husband of Mrs. C. (who has suffered for months from over-eating) hears the news. If, however, Mr. C. is card-indexed by the State to attend a doctor other than Q., he continues to disembogue for many months before he is allowed to change his doctor. No matter how inconvenienced Mr. C. may be, should he represent the low type of higher animal who would rather die than be muzzled in an infected area, such an exigency would not, of course, be provided for in any Act of Parliament.

Yet the proof of any system of health control is its pliability in emergency. Just how much this will affect the medical profession is hard to say, but a continued movement in the direction of complete State control would be likely to put the family doctor farther along the road to Carey Street. Looking back, his view must be that the Lord sent the patient, but the Government has taken him away again; and a system of further control on State lines would seem to suggest that it will not be the patient who has eventually to be rescued from penury. Nowadays one seldom hears of a panel doctor pulling down his hen-house so as to build a motor-garage. Possibly the golden eggs are all in the State basket.

Only with much inherited perspicacity and a further acquiring of clairvoyant proclivity could Dr. Addison, from a distance, be expected to differentiate between the man in the street and the individual who prefers to walk on the pavement. His desire for a panel system that will rope in a larger part of the population may also include those who are requested to keep off the grass. It would seem that he is impelled to take certain measures through this lack of recognition. But what is good for the goose, as every doctor knows, is not always good for the gander. Mayhap the mind of the Minister of Health is hermaphrodically made up. That will soon be the doctor's secret.—I am, Sir, yours faithfully,

January 25, 1921. H. L. CROXLEY.

### Entire State Control.

*To the Editor of* THE HOSPITAL.

